# *Bacillus velezensis* T971 genome informs starch degradation in tobacco

**DOI:** 10.3389/fmicb.2025.1689015

**Published:** 2025-11-26

**Authors:** Liwei Hu, Xinpeng Zhang, Qin Gao, Mengmeng Yang, Xiangzhou Dong, Tingming Cheng, Taibo Liang, Bingye Yang, Yanling Zhang, Yanbin Yin

**Affiliations:** 1 Zhengzhou Tobacco Research Institute of CNTC, Zhengzhou, China; 2 Department of Food Science and Technology, University of Nebraska-Lincoln, Lincoln, NE, United States; 3 Anhui Wannan Tobacco Co. Ltd., Xuancheng, China

**Keywords:** starch, *Nicotiana tabacum*, *Bacillus velezensis*, amylase, CAZyme

## Abstract

Starch is an important reserve polysaccharide in tobacco leaves. An endophytic bacterium, *Bacillus velezensis* T971, was isolated from the leaves of *Nicotiana tabacum* L. Yunyan 97 and showed strong starch-degrading activity on the agar plate containing 1% starch (w/v). The complete genome of T971 was determined using PacBio long-read sequencing technology, revealing a single circular chromosome of 3,930,941 bp encoding 3,692 proteins. The T971 genome was compared with 242 other complete genomes of *B. velezensis*. Pan-genome analysis revealed a sporadic distribution of the plantazolicin (PZN) biosynthetic gene cluster (BGC) and mobile genetic elements (MGEs) (e.g., genomic islands (GIs) and prophages), which have contributed to the variability of *B. velezensis* genomes. Carbohydrate-active enzyme (CAZyme) analysis found 113 CAZymes in the T971 genome, including 41 glycoside hydrolases (GHs), 40 glycosyltransferases (GTs), and 14 carbohydrate esterases (CEs). One (GH13_28 family) of the four *α*-amylases is the most promising candidate for starch degradation in tobacco leaves, possessing a signal peptide and two carbohydrate-binding modules (CBMs). This study identifies *B. velezensis* T971 as a potential strain for industrial amylase production.

## Introduction

Starch is an important reserve polysaccharide present in tobacco (*Nicotiana tabacum*) leaves (Ancín et al., 2019; Chen et al., 2019). Starch can be degraded by amylases to produce glucose, maltose, and dextrin (Farooq et al., 2021). Amylases are carbohydrate-active enzymes (CAZymes) that catalyze the hydrolysis of *α*-1,4 glycosidic bonds and can be classified into several types, including α-amylases, β-amylases, and *γ*-amylases (Akinfemiwa et al., 2024; Farooq et al., 2021). Microbial amylases are particularly important in the food industry (Mehta and Satyanarayana, 2016). Given that numerous microorganisms inhabit tobacco plants, researchers have shown great interest in identifying and isolating amylase-producing bacteria from tobacco leaves. The reason is that these bacteria can be used for the microbial fermentation of tobacco leaves (Ma et al., 2024; Wen et al., 2023; Zhang et al., 2024a).

For example, *Bacillus kochii, Paenibacillus amylolyticus,* and *Bacillus subtilis* were recently shown to effectively reduce the starch content of tobacco leaves during the curing stage (Gong et al., 2023; Wu et al., 2022; Zhang et al., 2024a). The thermophilic strain *B. subtilis* ZIM3 was also found to simultaneously degrade both starch and cellulose in tobacco leaves across a wide range of temperatures and pH values (Dai et al., 2020). However, few microbes were shown to degrade starch during the field growth stage of tobacco plants (Hu et al., 2022).

In this study, we report the isolation of an endophytic bacterium, *Bacillus velezensis* T971, from the tobacco leaves of *Nicotiana tabacum* L. Yunyan 97. Our results demonstrated that T971 can degrade starch in Yunyan 97 leaves during the field growth stage. The complete genome of T971 was determined using PacBio long-read DNA sequencing technology. Genome analysis identified amylases and other carbohydrate-degrading CAZymes. Pan-genome analysis revealed that *B. velezensis* T971 (GCA_041893155.1) is closely related to UFLA258 (GCF_004799565.1). This study enhances our understanding of the molecular mechanism underlying starch degradation by tobacco endophytes.

## Materials and methods

### Isolation of *Bacillus velezensis* strain T971

Leaves of *N. tabacum* L. Yunyan 97, 50 days old, were collected and washed with 70% ethanol for 5 min. Then, the leaves were washed with 0.9% sodium hypochlorite for 20 min and further washed three times with sterile water, following a previous report (Njoloma, 2023). The washed leaves were cut into tissue blocks of 5 × 5 mm on a cleaning bench and rinsed in sterile water for 5 min. The eluent was spread onto a 1% starch-containing nutrient agar (NA) plate and incubated at 25 °C for 48 h-60 h. The cultured plates were treated with 1% iodine solution for 10 min, followed by decolorization with distilled water. A single bacterial colony with an obvious hydrolysis circle was picked and re-lined three times. A newly grown single colony was purified as the *B. velezensis* T971 bacterial strain. The purified colonies were inoculated into LB liquid medium at 37 °C for 24 h, then mixed with the bacterial fermentation broth with 25% glycerol solution and stored in a − 80 °C freezer. The *B. velezensis* T971 culture was deposited in the China Center for Type Culture Collection (CCTCC) under the accession number: CCTCCM20232626.

### Molecular identification of T971

The T971 fermentation broth was centrifuged at 8,000 rpm for 10 min. Bacterial pellets were obtained for genomic DNA extraction using the Wizard genomic DNA purification kit (Promega, USA) according to the manufacturer’s standard protocol. DNA quality was verified by electrophoresis, and the universal 16S rDNA primers 27F and 1492R were used for PCR amplification with Taq DNA Polymerase (Sangon Biotech Shanghai, Co., Ltd). The amplification procedure began with a pre-denaturation at 94 °C for 5 min, followed by 35 cycles of denaturation at 94 °C for 30s, annealing at 58 °C for 30s, and extension at 72 °C for 90s, with a final extension at 72 °C for 10 min. PCR products were analyzed by 1% agarose gel electrophoresis and sequenced using the Sanger method. The 16S rRNA sequence was searched in GenBank using BLASTN.

### Genome sequencing of T971

Genomic DNA from *B. velezensis* T971 was extracted using the DNeasy UltraClean/NoviPure Microbial Kits (QIAGEN, Germany) according to the manufacturer’s standard protocol and quantified using a Qubit fluorometer. The integrity of the genomic DNA was checked by agarose gel electrophoresis.

Genomic DNA was sheared into selected fragments ranging from 6 kb to 20 kb using the Covaris g-TUBE device (Woburn, USA) and size-selected with the BluePippin fragment (Sage Science, USA). For PacBio long-read sequencing, the SMRTbellTM Template Kit (version 2.0) was used to construct SMRTbell libraries. The constructed library was quantified using a Qubit fluorometer and sequenced using PacBio Sequel II (Pacific Biosciences, USA) at Beijing Novogene Co., Ltd. in China.

For Illumina short-read sequencing, the DNA samples that passed the electrophoresis test were sheared into fragments of ~350 bp using a Covaris ultrasonic crusher. After processing the DNA fragments, the entire library was prepared using the NEBNext®Ultra™ DNA Library Prep Kit. The library was quantified using Qubit 2.0 and diluted to 2 ng/սL, and the inserts of the library were detected using the Agilent 2,100 tool and sequenced with NovaSeq PE150.

### Genome assembly and bioinformatics analysis of strain T971

The PacBio reads were used to assemble the draft genome of T971. The Illumina reads were used to polish the draft genome to obtain the final version of the complete genome. Specifically, the PacBio reads were first quality-filtered according to a recent study (Gao et al., 2024). The filtered reads were *de novo* assembled using Canu v2.0 into a single contig, which was polished with Racon v1.4.13 and Pilon v1.22 for three rounds of error correction (Koren et al., 2017; Vaser et al., 2017; Walker et al., 2014).

GeneMarkS-T v5.1, tRNAscan-SE v1.23, and RNAmmer v1.2 were used to predict protein-coding genes, tRNA genes, and rRNA genes, respectively (Besemer et al., 2001; Chan et al., 2021; Lagesen et al., 2007). Protein functional annotation was conducted by searching against the CAZy and COG databases (Galperin et al., 2021; Lombard et al., 2014). From the CAZy database, CAZymes involved in degrading starch, chitin, and cellulose were identified according to EC numbers from dbCAN3 search results (Zheng et al., 2023).

RepeatMasker (Version open-4.0.5) and Tandem Repeats Finder (TRF, Version 4.07b) were used for the prediction of interspersed nuclear elements and tandem repeats, respectively. Secondary metabolic gene clusters were predicted using antiSMASH version 4.0.2 (Medema et al., 2011). The identification, annotation, and visualization of prophages were performed using the PHASTEST web server (Wishart et al., 2023).

To compare our T971 genome (NCBI assembly ID GCA_041893155.1) with 242 publicly available *B. velezensis* genomes in GenBank, a pan-genome analysis was conducted using Anvio v8 (Eren et al., 2021). Single-copy core genes were identified from the pan-genome analysis, and their protein sequence alignment was used to build a species tree using GToTree (Lee, 2019). The tree was visualized using iTOL with default parameters (Letunic and Bork, 2024). Isolation sites and sources of these 242 genomes were curated from the NCBI BioSample database and compared with T971. The annotation of T971 and specific genes was performed using eggNOG (Huerta-Cepas et al., 2019).

## Results

### Isolation of *Bacillus velezensis* T971

**Figure 1:**
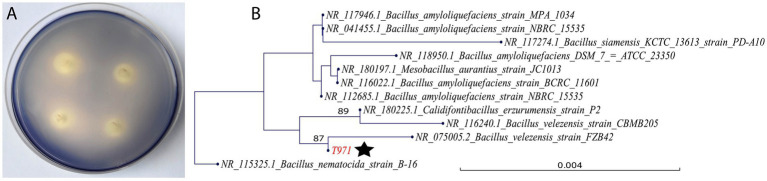
Characterization of T971. **(A)** Four colonies of the strain T971 grown on a nutrient agar (NA) plate containing 1% starch. **(B)** Phylogeny constructed with the 16S rRNA sequence of strain T971 (marked with a star) and its closest homologs from GenBank. The tree is rooted with B. nematocida strain B-16 (NR_115325.1) as the outgroup. A larger tree with more 16S rRNA sequences is provided in Supplementary Figure S1.

Strain T971 was isolated from 50-day-old Yunyan 97 leaves in the field and characterized as a *Bacillus velezensis* species by 16S rRNA sequencing. T971 grew well on plates containing 1% starch and exhibited a typical hydrolysis circle when iodine was added (Figure 1A). The ratio of the diameter of the hydrolyzed transparent circle to the diameter of the colony was 2.31, confirming its effective starch degradation ability. The phylogenetic tree of the 16S rRNA sequences (Figure 1B) showed that T971 was clustered with *B. velezensis* strains FZB42 (NR_075005.2) and CBMB205 (NR_116240.1), as well as *Calidifontibacillus erzurumensis* strain P2 (NR_180225.1).

### Genome sequencing of *Bacillus velezensis* T971

To better understand the genomic basis of T971 for starch degradation, we sequenced its complete genome. We obtained a total of 394,565 long reads with an average read length of 3,552 bp (Table 1). We assembled these reads, and the final genome of strain T971 was assembled as a single circular chromosome with a total length of 3,930,941 bp. Gene prediction found 3,692 protein-coding genes with an average gene length of 938 bp (Table 1). The genome has been submitted to NCBI with the genome assembly ID GCA_041893155.1 and the genome accession number CP169072.1.

**Table 1:** Genome sequencing statistics of *Bacillus velezensis* T971.

Features	Numbers
PacBio reads	394,565
Mean PacBio read length (bp)	3,552
N50 PacBio read length (bp)	4,195
Illumina paired-end read pairs	6,930,340
Illumina read length (bp)	150
Genome length (bp)	3,930,941
Genome GC%	46.63
Protein-coding genes	3,692

### Genome comparisons with other *Bacillus velezensis* genomes

**Figure 2:**
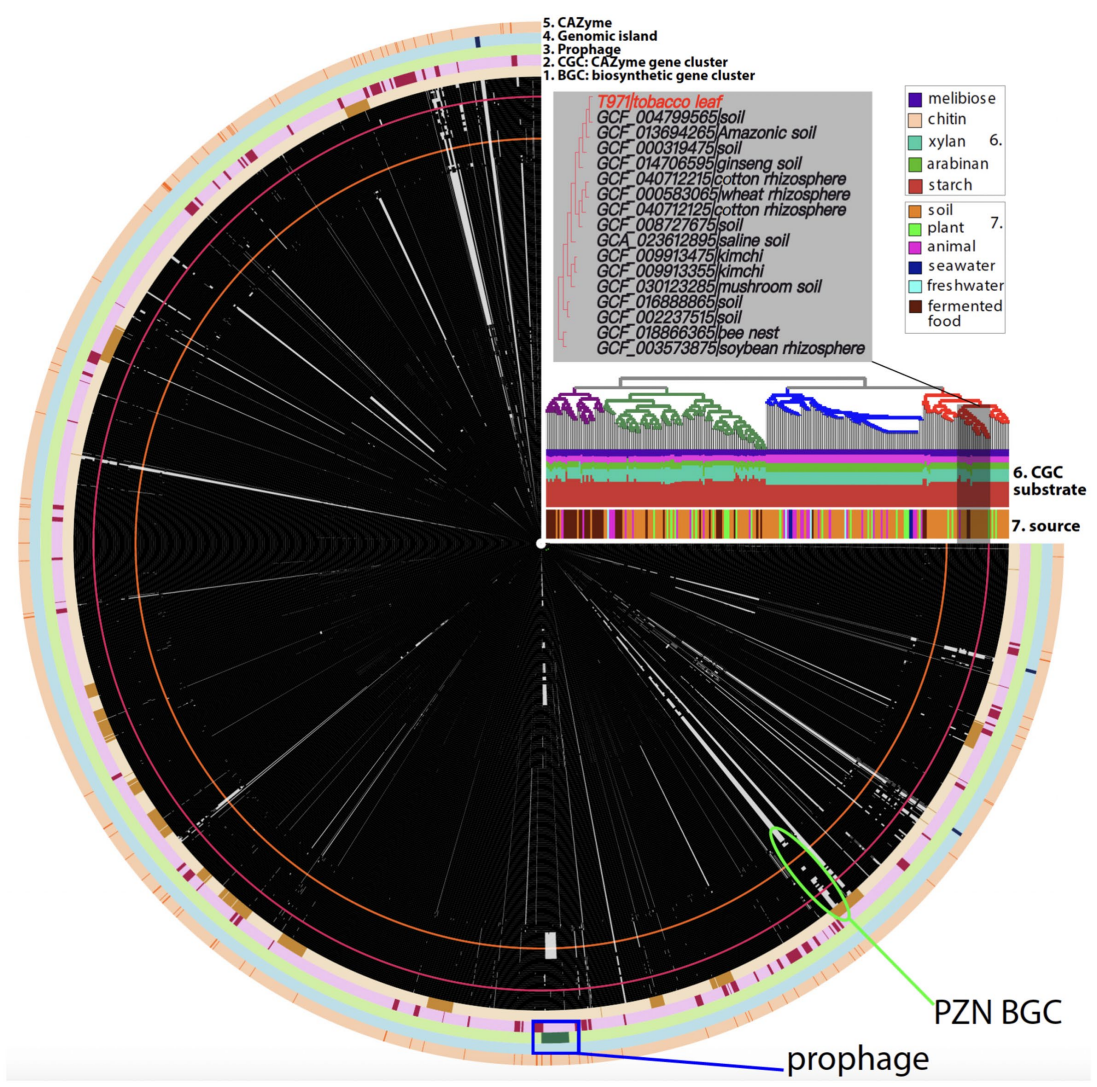
Anvio plot of the pan-genome analysis of 243 *Bacillus velezensis* genomes. From the innermost, each circle represents a genome. There are in total 243 circles, and each circle consists of protein families depicted as tiny black (present) or grey (absent) boxes. T971 (GCA_041893155.1) is shown as a red circle with red boxes. The black boxes indicate that the genome contains the protein family, and the grey boxes indicate that the genome does not contain the protein family. Only protein families present in T971 are plotted here. The protein families are arranged according to their positions in the T971 genome. A total of five outermost rings are shown: biosynthetic gene cluster (BGC), CAZyme gene cluster (CGC), prophage, genomic island, and CAZyme. CAZyme is for carbohydrate-active enzymes. The phylogeny was built using single-copy core genes in Anvio. Each branch of the tree aligns with a corresponding genome circle. In total, four major clades are indicated with different colors. An enlarged subtree with T971 is shown. Leaf labels are provided with the NCBI genome assembly IDs and isolation sources. The isolation sources for the whole tree are also summarized as a barplot (indicated as 7). Another stacked barplot (indicated as 6) is shown to indicate the predicted carbohydrate substrates of CGCs. The green oval indicates the location of the BGC for the synthesis of plantazolicin (PZN), which was first discovered in *Bacillus velezensis* FZB42 (Scholz et al., 2011) (orange circle). The blue rectangle indicates the location of the prophage in T971.

A keyword search of the GenBank database found 242 completely assembled *B. velezensis* genomes (as of August 2024), all representing single circular genomes without gaps. We compared the T971 genome with these publicly available genomes for a pan-genome analysis using Anvio (Figure 2). Single-copy core genes were identified in the 243 genomes, and a phylogenetic tree was built using the concatenated single-copy core gene alignment. The phylogeny revealed four major clades (Figure 2), one of which contains T971. According to the subtree, T971 is most closely related to *B. velezensis* strain UFLA258 (GCF_004799565.1) from soil (Figure 2). Other closely related genomes are also mostly from different *B. velezensis* strains associated with plants and soil, including the rhizosphere (Rabbee et al., 2019). This suggests that T971 is most likely derived from soil.

**Figure 3:**
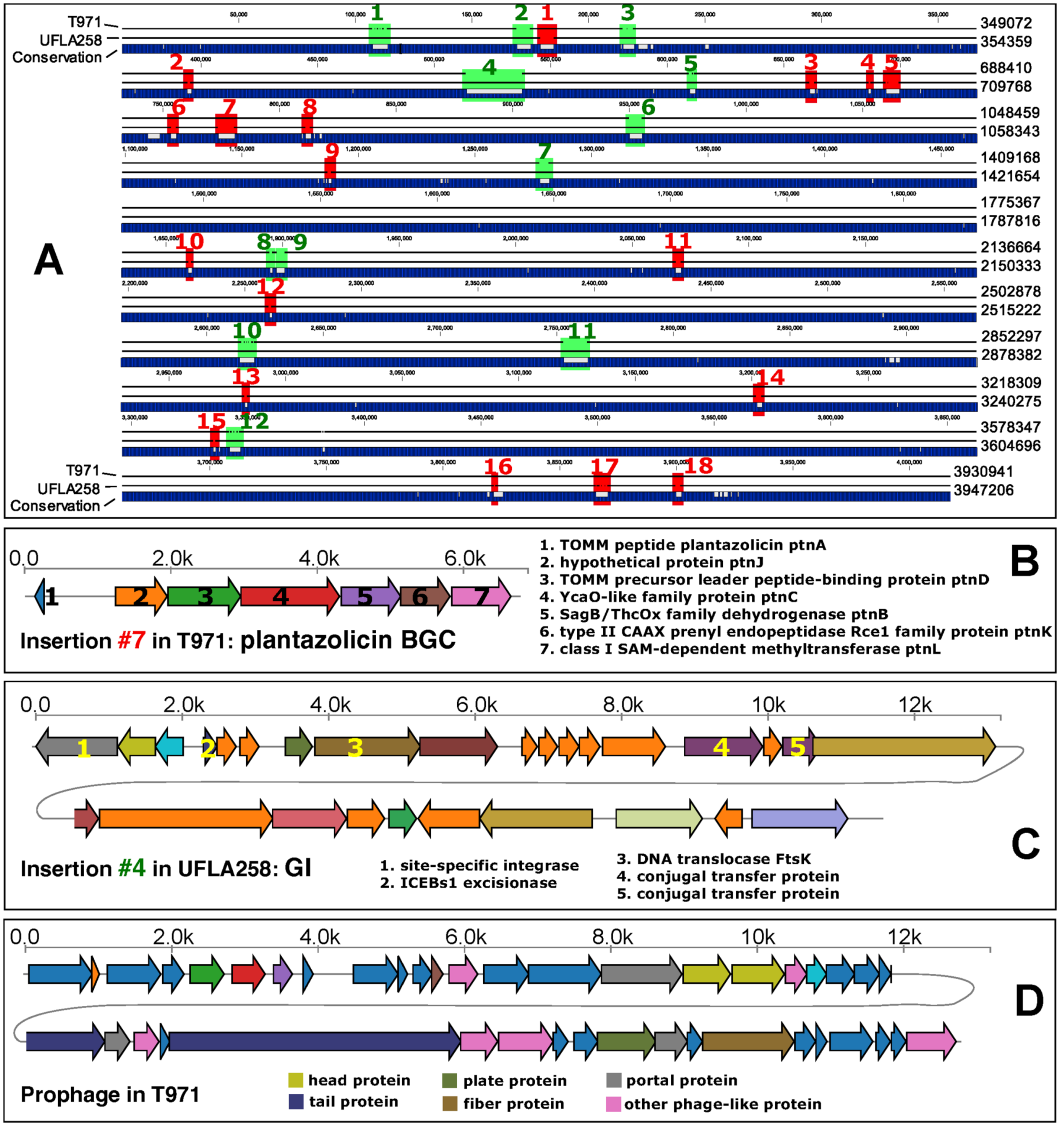
Comparative genomics of T971 and UFLA258. **(A)** Whole-genome alignment of the two genomes. Insertions larger than 1,000 bp are indicated with red (T971) and green (UFLA258) colors and labeled with numbers. Genome coordinates are shown on the right, without counting gaps. **(B)** Illustration of the genes in insertion #7 of T971 encoding a biosynthetic gene cluster (BGC) for plantazolicin synthesis. Functional descriptions are provided for all genes. **(C)** Illustration of the genes in insertion #4 of UFLA258 encoding a potential mobile genetic element (MGE). Functional descriptions are provided only for genes potentially responsible for gene transfer. **(D)** Illustration of the genes in the prophage of T971 (blue rectangle in Figure 2).

The average nucleotide identity between the T971 genome (length: 3,930,941 bp) and the UFLA258 genome (length: 3,947,206 bp) was 97.55%. With the whole-genome alignment (Figure 3A), we identified 18 large insertions (>1,000 bp) unique to T971 (relative to UFLA258, Supplementary Table S1) and 12 large insertions unique to UFLA258 (relative to T971, Supplementary Table S2). For example, insertion #7 in T971 corresponds to a known biosynthetic gene cluster (BGC0000569.5) in the MIBiG database (Zdouc et al., 2025); this BGC has seven genes (Figure 3B) responsible for the synthesis of plantazolicin (PZN), an antibiotic belonging to the thiazole/oxazole-modified microcin (TOMM) natural product class. PZN was first discovered in *B. velezensis* FZB42 (orange circle in Figure 2) (Scholz et al., 2011), and the BGC has also been identified in other Gram-positive soil bacteria (Molohon et al., 2011; Thanh Tam et al., 2023). Interestingly, a recent study found that the PZN BGC is only sporadically present in some, but not all, *B. velezensis* genomes (Thanh Tam et al., 2023). This finding is in agreement with our result that UFLA258 does not have this BGC (Figure 3A), which was also verified by an antiSMASH search (ring #1 in Figure 2) (Blin et al., 2023). In fact, most *B. velezensis* genomes included in our pan-genome analysis do not have the BGC (green oval in Figure 2). Horizontal gene transfer via genomic islands (GIs) is thought to be the reason for this sporadic distribution of the PZN BGC (Thanh Tam et al., 2023).

Another example is insertion #4 in UFLA258 (Figure 3C). This is the largest insertion with 26 protein-coding genes. This insertion was predicted to be a genomic island (GI) by IslandViewer (Bertelli et al., 2017), but it is not shown in Figure 2, as only protein families present in T971 are plotted (T971 has three GIs indicated as ring #4 in Figure 2). Indeed, half of the genes in insertion #4 have no known functions, and at least five genes (Figure 3C) encode DNA integrase, excisionase, translocase, and conjugal transfer proteins, which are key for horizontal gene transfer.

In addition to the genome comparison between T971 and UFLA258, we also searched for prophages and GIs in T971. A prophage of 31.9 kb, encoding 42 proteins, was detected in the T971 genome (Figure 3D). This prophage appears to be quite conserved across different *B. velezensis* genomes, but with variations (Figure 2). For example, 23 genes in the middle of the prophage are missing in the FZB42 genome and its closely related genomes, suggesting frequent gene loss and relaxed evolutionary selection on the prophage. A total of three GIs (ring #3 in Figure 2) were predicted in T971, with a total length of ~15 kb.

### Amylases and other CAZymes in the *Bacillus velezensis* T971 genome

The complete genome of T971 was annotated for CAZymes, including amylases, which were further compared with the CAZyme repertoires of closely related genomes. Using dbCAN3, we found 113 CAZymes (ring #5 in Figure 2; Supplementary Table S3), including 41 glycoside hydrolases (GHs), 40 glycosyltransferases (GTs), 14 carbohydrate esterases (CEs), five enzymes with auxiliary activities (AAs), three polysaccharide lyases (PLs), and 17 enzymes with carbohydrate-binding modules (CBMs). Interestingly, 13 of the 17 CBM-containing CAZymes have CBM50 domains, which are known to bind N-acetylglucosamines in the cell walls of bacteria and fungi. These CBM50 proteins (four with signal peptides) may play an important role for T971 to eliminate microbial competitors in the environment. CE4, CE14, GH23, and GH73, which are involved in the degradation of chitin and peptidoglycan in the cell walls of bacteria and fungi, have seven and three genes in T971, respectively. In addition to CAZymes involved in the degradation of microbial cell walls, T971 also has many GT enzymes for the synthesis of bacterial cell wall polysaccharides and exopolysaccharides, for example, 16 GT2, seven GT4, four GT119, and four GT51. These polysaccharides may help protect T971, enhancing its survival against attacks by phages and other competing microbes.

**Figure 4:**
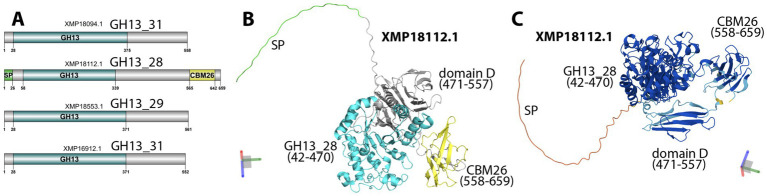
GH13 proteins in T971. **(A)** Functional domains in four GH13 proteins predicted by dbCAN3. **(B)** AlphaFold-predicted 3D structure of XMP18112 with colors matching those in **A**. **(C)** AlphaFold-predicted 3D structure of XMP18112 with colors representing the per-residue confidence predicted local distance difference test (plDDT) score, ranging between 0 (red) and 1 (blue). The XYZ axes in the 3D space are shown in the corners of **(B)** and **(C)**, respectively. The domain positions were determined by visually inspecting the 3D structural module boundaries. Domain D is named following AmyJ33r, as characterized in Montor-Antonio et al. (2017) and Hernández-Heredia et al. (2022).

More interestingly, the T971 genome also encodes CAZymes for plant polysaccharide degradation. In total, four GH13 proteins (Figure 4A) were found to degrade starch: two from the GH13_31 subfamily (EC 3.2.1.10 or 3.2.1.20, *α*-glucosidase), one from GH13_28 (EC 3.2.1.1, α-amylase), and one from GH13_29 (EC 3.2.1.93, trehalose-6-phosphate hydrolase). The GH13_28 protein (XMP18112) is particularly interesting as it also contains a signal peptide and a starch-binding CBM26 domain. The AlphaFold2-predicted structure of XMP18112 (Figure 4B) shows an unfolded N-terminal region, including the signal peptide, with low prediction scores (suggesting low foldability, Figure 4C), a distinctly folded GH13_28 domain (42–470), and a CBM26 domain (558–659). Between the two domains, there is also a well-folded, unannotated structural domain (471–557) consisting of β-sheets, commonly found in known CBMs (You et al., 2024). This unannotated structural domain corresponds to the domain D in the AmyJ33r protein (ANC5586.1) of *B. siamensis* JJC33M (Montor-Antonio et al., 2017). AmyJ33r has been biochemically characterized (Hernández-Heredia et al., 2022). It has the same sequence length (659 aa), the same modular domains, and shares 96% sequence identity with XMP18112 from T971, according to a sequence alignment of the two proteins (Supplementary Figure S2). AmyJ33r’s domains A, B, and C together (42–473) correspond to XMP18112’s GH13_28 domain and are essential for catalysis (Hernández-Heredia et al., 2022). In contrast, AmyJ33r’s domain D affects the enzyme’s efficiency at high pH, domain E (CBM26) binds to raw starch, and both domains determine the enzyme’s thermostability.

T971 also has GHs for other plant polysaccharide degradation: GH1 (β-glucosidase, EC 3.2.1.21), GH26 (β-glucosidase, EC 3.2.1.21; β-mannanase, EC 3.2.1.78), GH30 (β-xylanase, EC 3.2.1.8), GH32 (β-fructosidase, EC 3.2.1.80), GH4 (α-glucosidase, EC 3.2.1.20; α-galactosidase, EC 3.2.1.22), GH43 together with CBM91/CBM6 (α-arabinofuranosidase, EC 3.2.1.55; β-xylosidase, EC 3.2.1.37), GH51 (α-arabinofuranosidase, EC 3.2.1.55), GH16 (β-glucosidase, EC 3.2.1.6), GH5 together with CBM3 (β-glucanase, EC 3.2.1.6), GH68 (inulosucrase, EC 2.4.1.9), and others.

In addition to CAZymes, dbCAN3 also predicted CAZyme gene clusters (CGCs) and their glycan substrates (ring #2 in Figure 2; Supplementary Table S4). The largest substrate groups according to CGC predictions include starch, xylan, and arabinan, which are major polysaccharides in tobacco leaves.

## Discussion

*Bacillus velezensis* (formerly known as *B. amyloliquefaciens*) is a Gram-positive, rod-shaped bacterium belonging to the *B. subtilis* group (Abd El-Daim et al., 2019; Adeniji et al., 2019; Adeniji and Babalola, 2022, 2019). Previous reports suggest that *B. velezensis* strains are beneficial endophytes producing a range of bioactive compounds and enzymes, which show diverse abilities, including suppressing plant pathogens, promoting plant growth, and potentially mediating abiotic stress tolerance (Abd El-Daim et al., 2019; Feng et al., 2022; Rabbee et al., 2019; Yang et al., 2023; Zhou et al., 2022). For example, *B. velezensis* strain BR-01 can produce cellulase, β-1,3-glucanase, chitinase, indoleacetic acid, and siderophores and may produce three lipopeptide antibiotics—surfactin, iturin, and fengycin—which display strong antagonistic activities against a variety of rice pathogens (Zhou et al., 2022). *B. velezensis* strain WSW007 has the capacity to promote tobacco and tomato growth by producing beneficial volatiles, such as 2,3-butanediol and acetoin (He et al., 2024). Strain HY23 promotes the growth of soybeans under salt stress by producing exopolysaccharides (Zou et al., 2024). *B. velezensis* D103 isolated from maize can synthesize many enzymes, including amylase, cellulase, and β-1,3-glucanase, and it is also capable of nitrogen fixation, inorganic phosphorus solubilization, and potassium solubilization, which show significant growth stimulation (Zhang et al., 2024b).

We were interested in amylases encoded in *B. velezensis*. Previous studies have isolated amylase-producing *B. velezensis* strains from corn seeds (Hu et al., 2022), corn kernels (Nie et al., 2024), phenolic waste crystals (Chio et al., 2023), soils (Bhatt et al., 2020), and Daqu fermentation starters (Wang et al., 2024). In these studies, strain KB 2216 was shown to produce four different forms of amylases (Bhatt et al., 2020). In this study, we isolated strain T971 with amylase enzyme activity from tobacco in the field and identified it as *B. velezensis* based on 16S rRNA sequence BLAST analysis. We determined its complete genome and compared it with the genome of strain UFLA258. The genes for the biosynthesis of plantazolicin were first reported in 2008 (Lee et al., 2008). Plantazolicin has specifically been identified as a selective bactericidal agent (Fan et al., 2018). Insertion #7 in T971, relative to UFLA258, corresponds to a biosynthetic gene cluster containing seven genes responsible for the synthesis of plantazolicin, suggesting that strain T971 may have the ability to eliminate pathogens in tobacco.

*α*-amylases (EC 3.2.1.1) are key amylolytic enzymes found mainly in the CAZyme family GH13 (Janeček et al., 2014). Many α-amylases have three conserved structural domains (Janeček et al., 2014), while some α-amylases contain additional domains, usually located in their C-terminal region. For example, AmyJ33r of *B. siamensis* JJC33M has been shown to contain five domains (Montor-Antonio et al., 2017). The first three are required for catalysis, and the two C-terminal domains are important for binding to raw starch (CBM26) and controlling the enzyme’s efficiency and thermostability (Hernández-Heredia et al., 2022). T971 encodes a relatively large number of GH genes (41), including four α-amylases with GH13 domains. One of these α-amylases (XMP18112) also possesses a CBM26 domain and a signal peptide. From the AlphaFold2-predicted structure of XMP18112 (Figure 4), we also identified an unannotated structural domain rich in antiparallel β-sheets that may also be a CBM domain (You et al., 2024). Given the high sequence similarity between XMP18112 and AmyJ33r (Supplementary Figure S2), the two C-terminal CBM domains in XMP18112 must have the same functions as those in AmyJ33r (starch binding and enzyme stability). In other words, these CBMs may be critical and facilitate the binding and improve the degradation of starch in tobacco leaves (Majzlová and Janeček, 2014). Therefore, XMP18112 has great potential for industrial production of α-amylases. In addition to GHs, T971 also encodes 40 GTs, including 16 GT2, seven GT4, four GT119, and four GT51. Some of these GTs may be involved in the synthesis of bacterial cell wall polysaccharides and exopolysaccharides, which are critical for the survival of T971 and its ability to compete with other bacteria on tobacco leaves.

## Data Availability

The genome of *Bacillus velezensis* T971 is available in GenBank with a genome assembly ID GCA_041893155.1 and a genome accession number CP169072.1.
