# Supplementary figures and images for: Bacillus velezensis T971 genome informs starch degradation in tobacco
Source: Front Microbiol. 2025 Nov 26;16:1689015. doi: 10.3389/fmicb.2025.1689015 (PMC12689891; doi:10.3389/fmicb.2025.1689015)

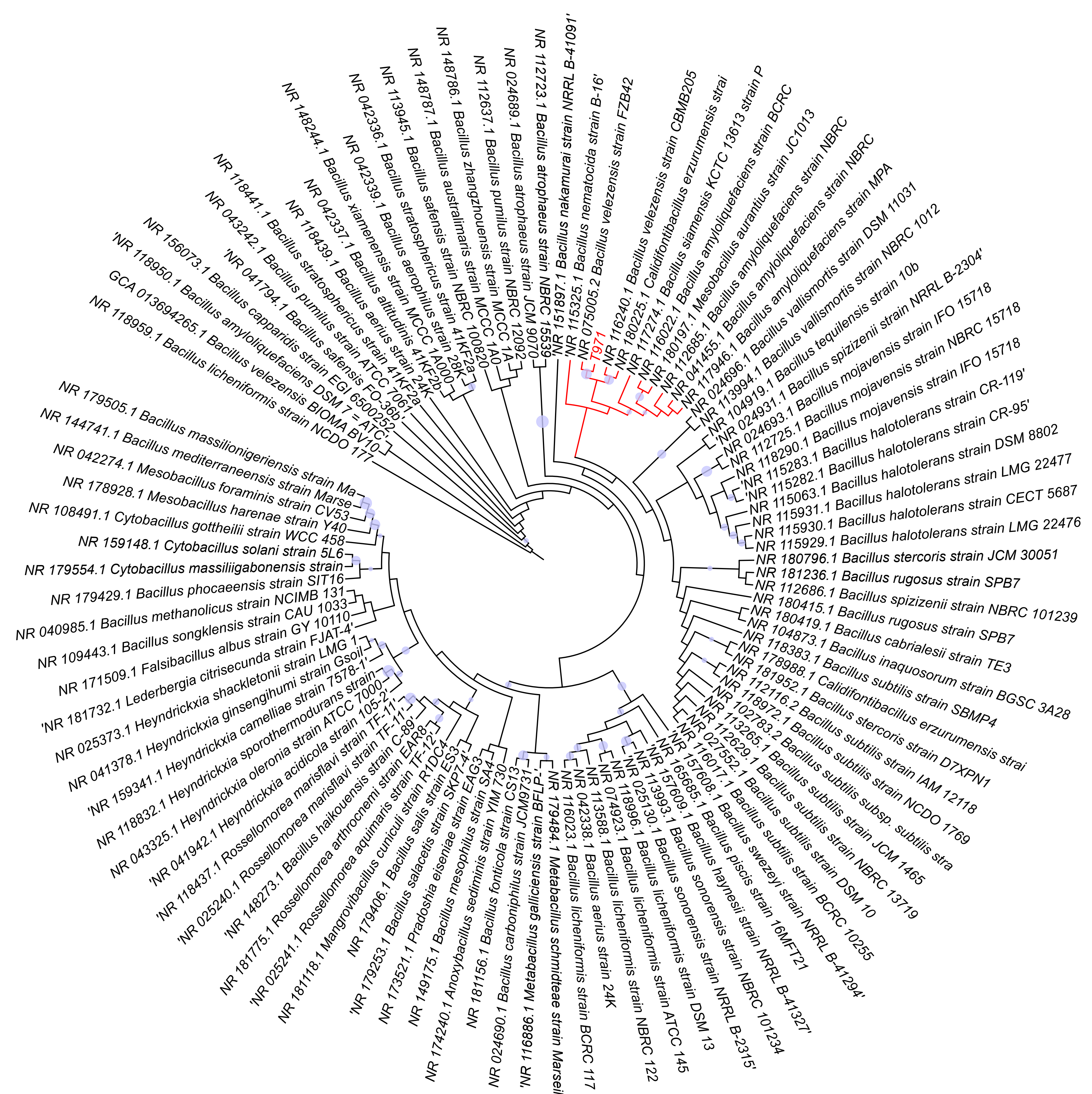

Supplement: Supplementary file 1 [file Image_1.pdf]

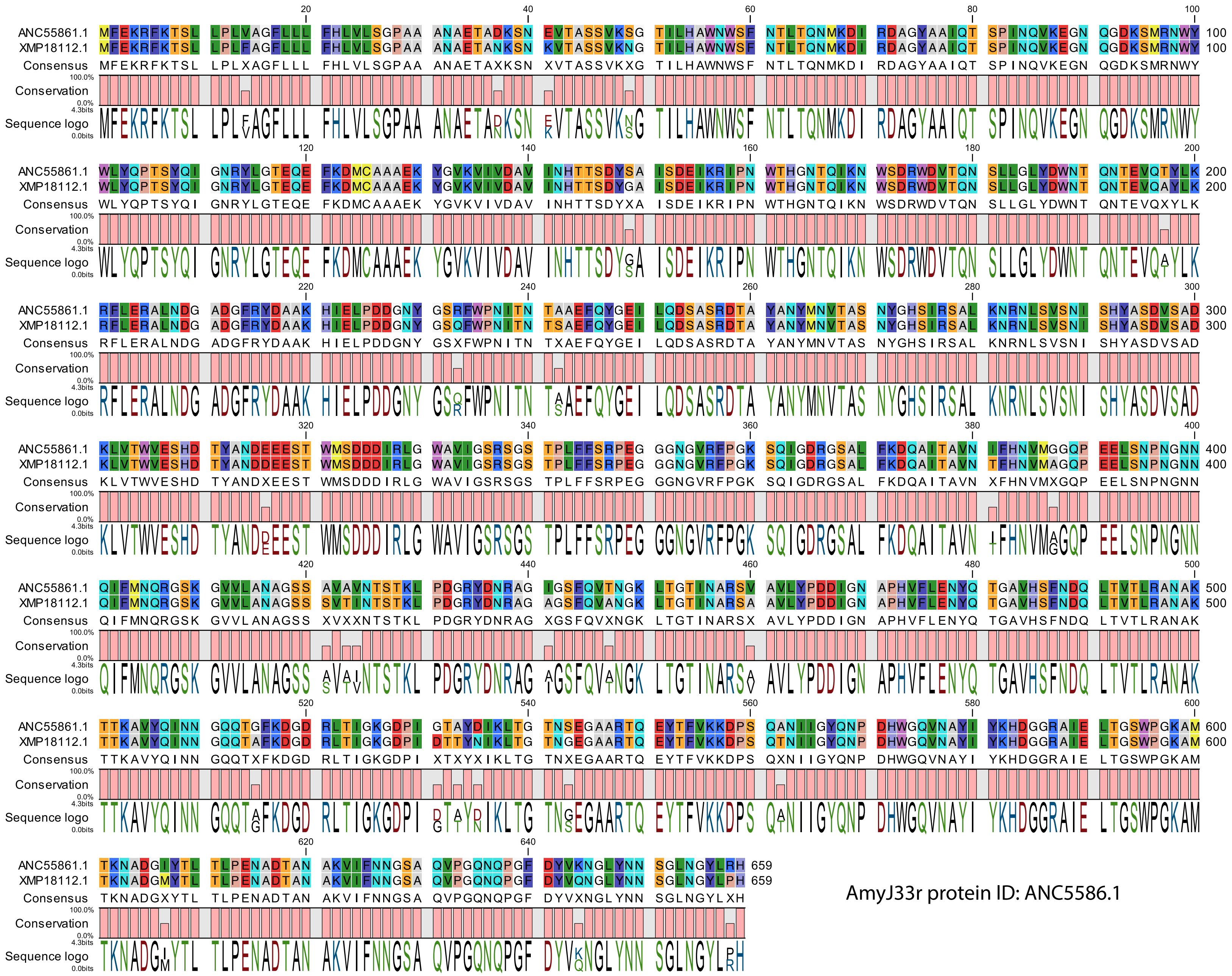

Supplement: Supplementary file 2 [file Image_2.pdf]
