# Supplementary material for: Bacillus velezensis T971 genome informs starch degradation in tobacco
Source: Front Microbiol. 2025 Nov 26;16:1689015. doi: 10.3389/fmicb.2025.1689015 (PMC12689891; doi:10.3389/fmicb.2025.1689015)
Supplement: Supplementary file 3 [file Table_1.docx]

**Table S1. Inserted regions (>1kb) encoding proteins in T971 relative to UFLA258**

| **insertion ID in T971*** | **gene ID** | **description** |
| --- | --- | --- |
| 12 | XMP16215.1 | IS3 family transposase |
| 14 | XMP17072.1 | SDR family NAD(P)-dependent oxidoreductase |
| 14 | XMP17073.1 | LysR family transcriptional regulator |
| 14 | XMP17074.1 | LysR family transcriptional regulator |
| 16 | XMP17666.1 | hypothetical protein ACETWW_18455 |
| 16 | XMP17667.1 | hypothetical protein ACETWW_18460 |
| 16 | XMP17668.1 | Imm26 family immunity protein |
| 16 | XMP17669.1 | hypothetical protein ACETWW_18470 |
| 16 | XMP17670.1 | DNRLRE domain-containing protein |
| 17 | XMP17700.1 | sensor histidine kinase |
| 17 | XMP17701.1 | response regulator transcription factor |
| 17 | XMP17702.1 | ABC transporter ATP-binding protein |
| 17 | XMP17703.1 | ABC transporter permease |
| 17 | XMP17704.1 | ABC transporter permease |
| 18 | XMP17731.1 | M48 family metalloprotease |
| 18 | XMP17732.1 | restriction endonuclease |
| 2 | XMP18163.1 | IS3 family transposase |
| 3 | XMP18398.1 | glycoside hydrolase domain-containing protein |
| 4 | XMP18420.1 | cation diffusion facilitator family transporter |
| 5 | XMP18426.1 | DEAD/DEAH box helicase family protein |
| 8 | XMP18441.1 | amidophosphoribosyltransferase |
| 7 | XMP18497.1 | TOMM peptide plantazolicin ptnA |
| 7 | XMP18498.1 | hypothetical protein ACETWW_03785 ptnJ |
| 7 | XMP18499.1 | TOMM precursor leader peptide-binding protein ptnD |
| 7 | XMP18500.1 | YcaO-like family protein ptnC |
| 7 | XMP18501.1 | SagB/ThcOx family dehydrogenase ptnB |
| 7 | XMP18502.1 | type II CAAX prenyl endopeptidase Rce1 family protein ptnK |
| 7 | XMP18503.1 | class I SAM-dependent methyltransferase ptnL |
| 9 | XMP18866.1 | class I SAM-dependent methyltransferase |
| 9 | XMP18867.1 | MFS transporter |
| 10 | XMP19428.1 | IS3 family transposase |
| 6 | XMP19578.1 | T7SS effector LXG polymorphic toxin |

* see red labeled regions in Figure 3A
