# Supplementary material for: Bacillus velezensis T971 genome informs starch degradation in tobacco
Source: Front Microbiol. 2025 Nov 26;16:1689015. doi: 10.3389/fmicb.2025.1689015 (PMC12689891; doi:10.3389/fmicb.2025.1689015)
Supplement: Supplementary file 4 [file Table_2.docx]

**Table S2. Inserted regions (>1kb) encoding proteins in UFLA258 relative to T971**

| **insertion ID in UFLA258*** | **gene ID** | **description** |
| --- | --- | --- |
| 1 | QCC34550.1 | mersacidin/lichenicidin family type 2 lantibiotic |
| 1 | QCC34551.1 | type 2 lantipeptide synthetase LanM |
| 1 | QCC34552.1 | ABC transporter ATP-binding protein |
| 1 | QCC34553.1 | hypothetical protein E4T61_00585 |
| 3 | QCC34647.1 | hypothetical protein E4T61_01155 |
| 3 | QCC34648.1 | hypothetical protein E4T61_01160 |
| 3 | QCC34649.1 | hypothetical protein E4T61_01165 |
| 3 | QCC34650.1 | hypothetical protein E4T61_01170 |
| 4 | QCC34912.1 | site-specific integrase |
| 4 | QCC34913.1 | ImmA/IrrE family metallo-endopeptidase |
| 4 | QCC34914.1 | XRE family transcriptional regulator |
| 4 | QCC34915.1 | ICEBs1 excisionase |
| 4 | QCC34916.1 | hypothetical protein E4T61_02595 |
| 4 | QCC34917.1 | DUF961 domain-containing protein |
| 4 | QCC34918.1 | DNA translocase FtsK |
| 4 | QCC34919.1 | replication initiation factor domain-containing protein |
| 4 | QCC34920.1 | hypothetical protein E4T61_02620 |
| 4 | QCC34921.1 | hypothetical protein E4T61_02625 |
| 4 | QCC34922.1 | hypothetical protein E4T61_02630 |
| 4 | QCC34923.1 | hypothetical protein E4T61_02635 |
| 4 | QCC34924.1 | conjugal transfer protein |
| 4 | QCC34925.1 | hypothetical protein E4T61_02645 |
| 4 | QCC34926.1 | conjugal transfer protein |
| 4 | QCC34927.1 | ATP-binding protein |
| 4 | QCC34928.1 | DUF1874 domain-containing protein |
| 4 | QCC34929.1 | hypothetical protein E4T61_02665 |
| 4 | QCC34930.1 | endopeptidase |
| 4 | QCC34931.1 | hypothetical protein E4T61_02675 |
| 4 | QCC34932.1 | cystatin-like fold lipoprotein |
| 4 | QCC34933.1 | hypothetical protein E4T61_02685 |
| 4 | QCC34934.1 | ATP-binding protein |
| 4 | QCC34935.1 | tetratricopeptide repeat protein |
| 4 | QCC34936.1 | hypothetical protein E4T61_02700 |
| 4 | QCC34937.1 | DUF3238 domain-containing protein |
| 5 | QCC35014.1 | MFS transporter |
| 5 | QCC35015.1 | Rrf2 family transcriptional regulator |
| 6 | QCC35289.1 | ABC transporter substrate-binding protein |
| 6 | QCC35290.1 | ABC transporter permease |
| 6 | QCC35291.1 | ABC transporter permease |
| 6 | QCC35292.1 | ABC transporter ATP-binding protein |
| 6 | QCC35293.1 | ABC transporter ATP-binding protein |
| 7 | QCC35618.1 | response regulator transcription factor |
| 7 | QCC35619.1 | HAMP domain-containing histidine kinase |
| 7 | QCC35620.1 | Mas-related G-protein coupled receptor member D |
| 7 | QCC35621.1 | IS3 family transposase |
| 7 | QCC35622.1 | sulfite exporter TauE/SafE family protein |
| 8 | QCC36125.1 | YafY family transcriptional regulator |
| 9 | QCC36128.1 | hypothetical protein E4T61_09095 |
| 9 | QCC36129.1 | hypothetical protein E4T61_09100 |
| 9 | QCC36130.1 | hypothetical protein E4T61_09105 |
| 9 | QCC36131.1 | hypothetical protein E4T61_09110 |
| 9 | QCC36132.1 | GNAT family N-acetyltransferase |
| 10 | QCC36765.1 | hypothetical protein E4T61_12485 |
| 10 | QCC36766.1 | hypothetical protein E4T61_12490 |
| 10 | QCC36767.1 | hypothetical protein E4T61_12495 |
| 10 | QCC36768.1 | DUF2188 domain-containing protein |
| 10 | QCC36769.1 | hypothetical protein E4T61_12505 |
| 10 | QCC36770.1 | hypothetical protein E4T61_12510 |
| 10 | QCC36771.1 | streptothricin N-acetyltransferase SatA |
| 11 | QCC36895.1 | hypothetical protein E4T61_13165 |
| 11 | QCC36896.1 | hypothetical protein E4T61_13170 |
| 11 | QCC36897.1 | hypothetical protein E4T61_13180 |
| 11 | QCC36898.1 | SAM-dependent methyltransferase |
| 11 | QCC36899.1 | type I restriction-modification system endonuclease |
| 12 | QCC37486.1 | DUF1433 domain-containing protein |
| 12 | QCC37487.1 | DUF1433 domain-containing protein |
| 12 | QCC37488.1 | lipase |

* see green labeled regions in Figure 3A
