# Supplementary material for: Bacillus velezensis T971 genome informs starch degradation in tobacco
Source: Front Microbiol. 2025 Nov 26;16:1689015. doi: 10.3389/fmicb.2025.1689015 (PMC12689891; doi:10.3389/fmicb.2025.1689015)
Supplement: Supplementary file 5 [file Table_3.docx]

**Table S3. CAZymes annotated in the T971 genome**

| Gene ID | CAZyme domains | Signal peptide |
| --- | --- | --- |
| XMP19468.1 | AA10 | SP |
| XMP16698.1 | AA4 | - |
| XMP18659.1 | AA6 | - |
| XMP18316.1 | AA7 | - |
| XMP18638.1 | AA7 | - |
| XMP16256.1 | CBM50 | - |
| XMP16617.1 | CBM50 | - |
| XMP16645.1 | CBM50 | - |
| XMP18964.1 | CBM50 | - |
| XMP18989.1 | CBM50 | - |
| XMP19001.1 | CBM50 | - |
| XMP19603.1 | CBM50 | - |
| XMP16044.1 | CBM50\|CBM50 | SP |
| XMP18697.1 | CBM50\|CBM50 | SP |
| XMP16067.1 | CBM50\|CBM50\|CBM50\|CBM50 | SP |
| XMP18692.1 | CBM50\|CBM50\|CBM50\|CBM50\|CBM50 | SP |
| XMP19044.1 | CBM50\|GH18 | - |
| XMP19554.1 | CBM50\|GH18 | - |
| XMP16529.1 | CE12 | SP |
| XMP16072.1 | CE14 | - |
| XMP16201.1 | CE14 | - |
| XMP17183.1 | CE14 | - |
| XMP16530.1 | CE4 | SP |
| XMP17636.1 | CE4 | - |
| XMP17995.1 | CE4 | - |
| XMP18574.1 | CE4 | SP |
| XMP18717.1 | CE4 | SP |
| XMP19177.1 | CE4 | - |
| XMP19377.1 | CE4 | SP |
| XMP16535.1 | CE6 | - |
| XMP18125.1 | CE7 | - |
| XMP17278.1 | CE9 | - |
| XMP16085.1 | GH1 | - |
| XMP17623.1 | GH1 | - |
| XMP17674.1 | GH1 | - |
| XMP18140.1 | GH1 | - |
| XMP18934.1 | GH1 | - |
| XMP17708.1 | GH109 | - |
| XMP17427.1 | GH11 | SP |
| XMP18227.1 | GH126 | SP |
| XMP18112.1 | GH13_28\|CBM26 | SP |
| XMP18553.1 | GH13_29 | - |
| XMP16912.1 | GH13_31 | - |
| XMP18094.1 | GH13_31 | - |
| XMP17651.1 | GH16_21 | SP |
| XMP18005.1 | GH171 | SP |
| XMP16038.1 | GH23 | - |
| XMP18884.1 | GH23 | - |
| XMP18988.1 | GH23 | - |
| XMP17626.1 | GH26 | SP |
| XMP18006.1 | GH3 | SP |
| XMP16084.1 | GH30 | SP |
| XMP19524.1 | GH30_8 | SP |
| XMP17243.1 | GH32 | - |
| XMP17569.1 | GH32 | - |
| XMP17797.1 | GH32 | - |
| XMP16837.1 | GH4 | - |
| XMP17610.1 | GH4 | - |
| XMP18588.1 | GH4 | - |
| XMP19454.1 | GH43_11\|CBM91 | - |
| XMP19525.1 | GH43_16\|CBM6 | SP |
| XMP17678.1 | GH43_4 | SP |
| XMP16708.1 | GH43_5 | SP |
| XMP17047.1 | GH46 | SP |
| XMP19520.1 | GH5_2\|CBM3 | SP |
| XMP16681.1 | GH51_1 | - |
| XMP16701.1 | GH51_1 | - |
| XMP18928.1 | GH53 | SP |
| XMP17796.1 | GH68_1 | SP |
| XMP16899.1 | GH73 | - |
| XMP17348.1 | GH73 | SP |
| XMP16068.1 | GT1 | - |
| XMP18366.1 | GT1 | - |
| XMP18946.1 | GT1 | - |
| XMP17419.1 | GT119 | - |
| XMP17576.1 | GT119 | - |
| XMP19196.1 | GT119 | - |
| XMP19231.1 | GT119 | - |
| XMP17224.1 | GT122 | - |
| XMP16109.1 | GT2 | - |
| XMP17221.1 | GT2 | - |
| XMP17223.1 | GT2 | - |
| XMP17226.1 | GT2 | - |
| XMP17328.1 | GT2 | - |
| XMP17344.1 | GT2 | - |
| XMP17353.1 | GT2 | - |
| XMP17558.1 | GT2 | - |
| XMP17563.1 | GT2 | - |
| XMP17588.1 | GT2 | - |
| XMP18230.1 | GT2 | - |
| XMP18508.1 | GT2 | - |
| XMP18514.1 | GT2 | - |
| XMP18620.1 | GT2 | - |
| XMP19010.1 | GT2 | - |
| XMP19035.1 | GT2 | - |
| XMP17346.1 | GT26 | - |
| XMP16146.1 | GT28 | - |
| XMP19232.1 | GT28 | - |
| XMP16200.1 | GT4 | - |
| XMP17225.1 | GT4 | - |
| XMP17227.1 | GT4 | - |
| XMP17327.1 | GT4 | - |
| XMP17332.1 | GT4 | - |
| XMP17783.1 | GT4 | - |
| XMP17786.1 | GT4 | - |
| XMP16186.1 | GT51 | - |
| XMP16936.1 | GT51 | - |
| XMP17511.1 | GT51 | - |
| XMP18763.1 | GT51 | - |
| XMP17602.1 | GT8 | - |
| XMP19009.1 | GT83 | - |
| XMP18524.1 | PL1_6 | SP |
| XMP17671.1 | PL1_8 | SP |
| XMP17298.1 | PL9_2 | SP |
